# Quantifying Chaos by Various Computational Methods. Part 1: Simple Systems

**DOI:** 10.3390/e20030175

**Published:** 2018-03-06

**Authors:** Jan Awrejcewicz, Anton V. Krysko, Nikolay P. Erofeev, Vitalyj Dobriyan, Marina A. Barulina, Vadim A. Krysko

**Affiliations:** 1 Department of Automation, Biomechanics and Mechatronics, Lodz University of Technology, 1/15 Stefanowski St., 90-924 Lodz, Poland; 2 Cybernetic Institute, National Research Tomsk Polytechnic University, 30 Lenin Avenue, 634050 Tomsk, Russia; 3 Department of Applied Mathematics and Systems Analysis, Saratov State Technical University, 77 Politechnicheskaya, 410054 Saratov, Russia; 4 Department of Mathematics and Modeling, Saratov State Technical University, 77 Politechnicheskaya, 410054 Saratov, Russia; 5 Precision Mechanics and Control Institute, Russian Academy of Science, 24 Rabochaya Str., 410028 Saratov, Russia

**Keywords:** Lyapunov exponents, Wolf method, Rosenstein method, Kantz method, neural network method, method of synchronization, Benettin method, Fourier spectrum, Gauss wavelets

## Abstract

The aim of the paper was to analyze the given nonlinear problem by different methods of computation of the Lyapunov exponents (Wolf method, Rosenstein method, Kantz method, the method based on the modification of a neural network, and the synchronization method) for the classical problems governed by difference and differential equations (Hénon map, hyperchaotic Hénon map, logistic map, Rössler attractor, Lorenz attractor) and with the use of both Fourier spectra and Gauss wavelets. It has been shown that a modification of the neural network method makes it possible to compute a spectrum of Lyapunov exponents, and then to detect a transition of the system regular dynamics into chaos, hyperchaos, and others. The aim of the comparison was to evaluate the considered algorithms, study their convergence, and also identify the most suitable algorithms for specific system types and objectives. Moreover, an algorithm of calculation of the spectrum of Lyapunov exponents based on a trained neural network has been proposed. It has been proven that the developed method yields good results for different types of systems and does not require a priori knowledge of the system equations.

## 1. Introduction

The first part of the present work is focused on the numerical investigation of classical dynamical systems to estimate velocity of divergence of the neighborhood trajectories with the help of a measure coupled with the Kolmogorov entropy [1] (or metrics). In reference [1], based on the mathematical results of Oseledec [2] and Pesin [3], it has been shown that the numerically imposed relations can be treated as exact/true values. The method proposed by Wolf [1] is most widely used to verify and study chaotic dynamics. However, also the Rosenstein [4] and Kantz [5] methods are often employed to estimate the largest Lyapunov exponents. The state-of-the-art of papers devoted to the theoretical background of the Lyapunov exponents and methods of their computations has been carried out by Awrejcewicz et al. [6]. In particular, the method of the choice of an embedding dimension has been described. The method of the correlating dimension, the false nearest neighbor method and the method of gamma-test have been presented based on the Hénon and Lorenz attractors. In particular, the occurrence of high computational difficulties has been observed in the case of the Wolf method and its marginally successful employment to small values of the studied data.

To avoid the abovementioned drawbacks, a novel neural network-based algorithm to estimate the largest Lyapunov exponents by considering only one coordinate has been proposed. In reference [6] have reported the neural network algorithm for computation of a full spectrum of Lyapunov exponents. A comparison of the results obtained by Golovko with the exact values of the Lyapunov exponents of the Lorenz and Hénon systems have exhibited small errors.

In References [7,8], the method of largest Lyapunov exponent computation using the synchronization phenomena of identical systems has been proposed. A few types of coupling have been studied, depending on the type of the considered system. It has been pointed out that large computation time is required to achieve full synchronization.

The method proposed in References [9,10] is particularly suitable to study chaotic dynamics of continuous mechanical systems. It should be emphasized that, owing to the results published by the authors of the present paper, the analysis of nonlinear dynamics based on the estimation of the Lyapunov exponents yields a conclusion that the mentioned problems have not been satisfactorily solved yet [1,4,5,9,10].

More recently, Vallejo and Sanjuan [11,12] have studied the predictability of orbits in coupled systems by means of finite-time Lyapunov exponents. This approach has allowed them to estimate how close the computed chaotic orbits are to the real/true orbits, being characterized by the systems shadowing properties.

In the present paper, classical systems (Hénon map [13], hyperchaotic Hénon map [14], logistic map [15], Rössler attractor [16], and Lorenz attractor [17]) were analyzed with Gauss wavelets [18], Fourier spectra and Poincaré maps of a chaotic attractor [19,20,21].

It is known that the fundamental property of chaos is the existence of strong sensitivity to a change of the initial conditions. The definition of chaos, given first by Devaney in 1989 [22], includes three fundamental parts. In addition to sensitivity to the variation of the initial conditions, a condition of mixing, known also as the transitivity condition and the regularity condition, measured by the density of the periodic points or classical notion of periodicity is also included. In 1992, Banks et al. [23] proved that the condition of sensitivity to the initial condition can be neglected, i.e., conditions of transitivity and periodicity imply the sensitivity condition.

Knudsen [24] has defined chaos as a function given on a bounded metric space which has a dense orbit and essentially depends on initial conditions.

Owing to the definition proposed by Gulick [25], chaos exists when either there is essential dependence on the initial conditions or a chaotic function has positive Lyapunov exponents in each point of the space and which does not eventually tend to a periodic orbit. This definition has been also employed in the present work.

## 2. Lyapunov Exponents

### 2.1. The Largest Lyapunov Exponent

The following dynamical system was considered:

(1) $$\dot{x}=f\left(x\right)$$

where *x* stands for the *N*-dimensional state vector.

Two closed phase points *x*_1_ and *x*_2_ were chosen (in the phase space). They stand for the origins of the trajectories $(x_{1}\left(t\right)$ and $x_{2}\left(t\right))$. The change in the distance *d* between two corresponding points of these trajectories under evolution of system (1) can be monitored by:

(2) $$d\left(t\right)=\left|\vec{\varepsilon }\left(t\right)\right|=\left|x_{2}\left(t\right)-x_{1}\left(t\right)\right|$$

If the dynamics of system (1) is chaotic, *d*(*t*) increases exponentially in time, i.e.,:

(3) $$d\left(t\right)\approx d\left(0\right)e^{kt}$$

This yields the average velocity of the exponential divergence of the trajectories:

(4) $$k\approx \frac{\ln\left[\frac{d\left(t\right)}{d\left(0\right)}\right]}{t}$$

or more precisely:

(5) $$k=\underset{\begin{array}{c} d\left(0\right)\to 0\\ t\to \infty \end{array}}{\lim}\frac{\ln\left[d\left(t\right)/d\left(0\right)\right]}{t}$$

The quantity *h* is known as the Kolmogorov-Sinai entropy (KS-entropy). Employing the KS-entropy, one can define the studied process, i.e., quantify if the process is regular or chaotic. In particular, if the system dynamics is periodic or quasi-periodic, the distance *d*(*t*) is not inversed in time and the KS-entropy is equal to zero (*h* = 0). If the system dynamics tends to a stable fixed point *d*(*t*) → 0, then *h* < 0. Contrarily, KS-entropy is positive (*h* > 0) if one deals with chaotic dynamics. KS-entropy is the maximum characteristic Lyapunov exponent that enables one to follow velocity of information lost with respect to the initial system state.

### 2.2. Results

The spectrum of Lyapunov exponents makes it possible to qualitatively quantify a local property with respect to the stability of an attractor. Consider a phase trajectory *x*(*t*) of the dynamical system (1), starting from the point *x*(0) as well as its neighborhood trajectory $x_{1}\left(t\right)$ as follows:

(6) $$x_{1}\left(t\right)=x\left(t\right)+\vec{\varepsilon }\left(t\right)$$

The following function can be constructed:

(7) $$\lambda \left[\vec{\varepsilon }\left(0\right)\right]=\underset{t\to \infty }{\lim}\frac{\ln\left[\frac{\left|\vec{\varepsilon }\left(t\right)\right|}{\left|\vec{\varepsilon }\left(0\right)\right|}\right]}{t}$$

which is defined on the vector of initial displacement $\vec{\varepsilon }\left(0\right)$ such that $\left|\vec{\varepsilon }\left(0\right)\right|=\varepsilon$, where *ε* → 0.

All possible rotations of the initial displacements vector with respect to *n* directions of the *N*-dimensional phase space of the Function (7) will suffer the jump-like changes in the finite series of the values $\lambda_{1},\lambda_{2},\lambda_{3},\ldots ,\lambda_{n}.$ These values of the function λ are called Lyapunov exponents (LEs). Positive/negative values of LEs can be viewed as a measure of the averaged exponential divergence/convergence of the neighborhood trajectories.

A sum of LEs stands for an averaged divergence of the phase trajectories flow. In the case of a dissipative system, i.e., a system possessing an attractor, this sum is always negative. As numerical case studies show, in some dissipative systems the LEs are invariant with respect to the chosen initial conditions. Hence, a spectrum of LEs can be understood as the property of an attractor.

Usually, LEs are presented in a sequence of LE values in decreasing order. For instance, symbols (+, 0, −) mean that for the analyzed attractor, there is one direction in a 3D space, where exponential stretching is exhibited, the second direction indicates neutral stability, and the third one—exponential compression. It should be noted that all attractors different from stable stationary points always have at least one LE equal to zero (in average sense, all points of a trajectory are bounded by a compact manifold and they cannot exhibit divergence or converge).

In what follows, relationships between the Lyapunov exponents and the properties and types of attractors are illustrated and discussed:

(1) *n* = 1. In this case only a stable fixed point can be an attractor (node or focus). There exists one negative LE denoted by $\lambda_{\;1}=(-),$
(2) *n* = 2. In 2D systems, there are two types of attractors: stable fixed points and limit cycles. The corresponding LEs follow:
  - $\left(\lambda_{1},\lambda_{2})=(-,-\right)$—stable fixed/fixed point;
  - $\left(\lambda_{1},\lambda_{2})=(0,-\right)$—stable limit cycle (one exponent is equal to zero).
(3) *n* = 3. In 3D phase space, there exist four types of attractors: stable points, limit cycles, 2D tori and strange attractors. The following set of LEs characterizes possible dynamical situations to be met:
  - $\left(\lambda_{1},\lambda_{2},\lambda_{3})=(-,-,-\right)$—stable fixed point;
  - $\left(\lambda_{1},\lambda_{2},\lambda_{3})=(0,-,-\right)$—stable limit cycle;
  - $\left(\lambda_{1},\lambda_{2},\lambda_{3})=(0,0,-\right)$—stable 2D tori;
  - $\left(\lambda_{1},\lambda_{2},\lambda_{3})=(+,0,-\right)$—strange attractor.

In the majority of the studied problems, it is impossible to give an analytical definition of LEs, since the analytical solution to the governing differential equations would have to be known. However, there exist reliable algorithms to find all Lyapunov exponents numerically. 

## 3. Methods of Analysis of Lyapunov Exponents

### 3.1. Benettin Method

We began with the numerical investigation of the Kolmogorov entropy of the Hénon-Heiles model. Numerical computations were carried out with accuracy up to 14 digits by means of employing the so-called method of central points. Observe that a similar method has been used in reference [26].

Based on the Lyapunov exponents, the ergodic properties of dissipative dynamical systems with a few degrees of freedom were numerically studied with the Lorenz system. The system exhibited the exponents spectrum of the (+, 0, −) type, and the exponents had the same values for the orbits beginning from an arbitrary point on the attractor. It means that the ergodic property of a general dynamical system can be quantified by a spectrum of the characteristic Lyapunov exponents. Below, a brief description of the used method is presented.

Let a point $x_{0}$ belong to the attractor *A* of a dynamical system. An evolution trajectory of the point $x_{0}$ is referred to as a real/true trajectory. A positive quantity ε, being significantly less than the attractor dimension, is chosen. Furthermore, an arbitrary perturbed point ${\tilde{x}}_{0}$ is chosen in a way to satisfy $\left\|{\tilde{x}}_{0}-x_{0}\right\|=\varepsilon .$ The evolution of points $x_{0}$ and ${\tilde{x}}_{0}$ is considered in a short time interval *T*, and new points are denoted by $x_{1}$ and ${\tilde{x}}_{1},$ respectively. A vector $\Delta x_{1}={\tilde{x}}_{1}-x_{1}$ is called the perturbation vector. The first estimate of the exponent is found with the use of the following formula

(8) $${\tilde{\lambda }}_{1}=\frac{1}{T}\ln\frac{\left||\Delta x_{1}|\right|}{\varepsilon }.$$

The time interval *T* is chosen in a way to keep the amplitude of perturbation less than the linear dimensions of the phase space nonhomogenity and the attractor dimension. The normalized perturbation vector $\Delta x_{1}^{\prime }=\varepsilon \Delta x_{1}/\left||\Delta x_{1}|\right|$ is taken, and a new perturbed point ${\tilde{x}}_{1}^{\prime }=x_{1}+\Delta x_{1}^{\prime }$ is defined. Finally, the so far described procedure is implemented taking into account $x_{1}$ and ${\tilde{x}}_{1}$ instead of $x_{0}$ and ${\tilde{x}}_{0}$, respectively.

After repeating this procedure *M* times, λ is defined as an arithmetic average of the estimates ${\tilde{\lambda }}_{l}$ obtained on each computational step:

(9) $$\lambda ≅\frac{1}{M}\sum_{i=1}^{M}{\tilde{\lambda }}_{l}=\frac{1}{M}\sum_{i=1}^{M}\frac{1}{T}\ln\frac{\left||\Delta x_{i}|\right|}{\varepsilon }=\frac{1}{MT}\sum_{i=1}^{M}\ln\frac{\left||\Delta x_{i}|\right|}{\varepsilon }.$$

In order to achieve a higher estimate, one can take large *M* and carry out computations for a different initial point $x_{0}$. This method can be used when the equations governing the system evolution are known. It should be noted, however, that these equations are usually unknown for the experimental data.

To compute the Lyapunov spectrum numerically, one can use another approach generalizing the Benettin’s algorithm. In general, it is necessary to follow a few trajectories of the perturbed points instead of only one, fundamental trajectory (the number of perturbed trajectories is equal to the dimension of the phase space). For this purpose, a numerical approach based on derivation of the dynamic equations in variations can be used [27]. Since the largest LE plays a crucial role in the evolution of all perturbed trajectories, it is necessary to carry out orthogonalization of the perturbation vectors on each step of the algorithm. In what follows, a procedure of numerical estimation of the Lyapunov spectrum of a dynamical system is briefly described. To simplify, the considerations are limited to 3D systems.

Let $r_{0}$ stand for a point of the chaotic attractor and *ε* be a fixed positive number, small in comparison to linear dimensions of the attractor. The points $x_{0}$, $y_{0}$ and $z_{0}$ are chosen so that the perturbation vectors $\Delta x_{0}=x_{0}-r_{0},\;\Delta y_{0}=y_{0}-r_{0},\;\Delta z_{0}=z_{0}-r_{0}$ have the length ε and are mutually orthogonal. After a certain small time interval *T*, the points $r_{0},x_{0},y_{0}$ and $z_{0}$ are transformed into points $r_{1},x_{1},\;y_{1}$ and $z_{1}$, respectively. Then, new perturbation vectors $\Delta x_{1}=x_{1}-r_{1},\;\Delta y_{1}=y_{1}-r_{1},\;\Delta z_{1}=z_{1}-r_{1}$ are considered. The orthogonlization using the well-known (in linear algebra) Gramm–Schmidt method is carried out. After this step, the obtained vectors of perturbation $\Delta x_{1}^{\prime \prime },\Delta y_{1}^{\prime \prime },\Delta z_{1}^{\prime \prime }$ become orthonormalized, i.e., they are mutually orthogonal and have the unit length. Then, the renormalization of the perturbation vectors is carried out again to get lengths of the vectors in terms of the magnitude *ε*:

(10) $$\Delta x_{1}^{\prime \prime \prime }=\Delta x_{1}^{\prime \prime }\times \varepsilon ,\;\;\;\Delta y_{1}^{\prime \prime \prime }=\;\Delta y_{1}^{\prime \prime }\times \varepsilon ,\;\;\Delta z_{1}^{\prime \prime \prime }=\Delta z_{1}^{\prime \prime }\times \;\varepsilon$$.

We take the following perturbed points:

(11) $$x_{1}^{\prime }=x_{1}+\;\Delta x_{1}^{\prime \prime \prime },\;\;y_{1}^{\prime }=y_{1}+\;\Delta y_{1}^{\prime \prime \prime },\;\;z_{1}^{\prime }=z_{1}+\;\Delta z_{1}^{\prime \prime \prime }$$

Next, the process is repeated, i.e., instead of the points $r_{0},x_{0},y_{0}$ and $z_{0}$, the points $r_{1},x_{1}^{\prime },y_{1}^{\prime }$ and $z_{1}^{\prime }$ are taken into account, respectively.

Repeating the so far described procedure *M* times, one finds:

(12) $$S_{1}=\sum_{k=1}^{M}\ln\|\Delta x_{k}^{\prime }\|,\;\;S_{2}=\sum_{k=1}^{M}\ln\|\Delta y_{k}^{\prime }\|,\;\;S_{3}=\sum_{k=1}^{M}\ln\|\Delta z_{k}^{\prime }\|.$$

Then, a spectrum $\Lambda =\left\{\lambda_{1},\lambda_{2},\lambda_{3}\right\}$ of LEs can be found by the following formulas:

(13) $$\lambda_{i}=\frac{S_{i}}{MT},\;\;i=1,\;2,\;3$$

In this method, the choice of time interval *T* is crucial. If one takes too large time interval *T*, then all perturbed trajectories are inclined in the direction corresponding to the maximum LE, and hence the obtained results are not reliable.

### 3.2. Wolf Method

In Reference [1], a novel algorithm to find nonnegative Lyapunov exponents by using a time series has been proposed. It has been illustrated that the Lyapunov exponents are associated with either exponential divergence or convergence of the neighborhood orbits in the considered phase space. In general, the method is applicable only when analytical governing equations are known, and it is based on tracing the large time-consuming increase in the number of elements in a small volume of an attractor.

We defined a Lyapunov exponent and a spectrum of Lyapunov exponents, and then illustrated how the system dynamics depends on the number of exponents with different signs in the spectrum. Our approach included reconstruction of an attractor and investigation of orbital divergence on the possibly smallest distances using the approximate Gramm–Schmidt orthogonalization procedure in the reconstructed phase. In order to estimate the largest Lyapunov exponent, a long trace of time evolution of the chosen pair of the neighborhood orbits was carried out. Note that a particular attention should be paid, since the reconstructed attractor may contain points belonging to different attractors. 

Two versions of the method are proposed. The first one includes the so-called fixed evolution time, where the time interval associated with the change of the points is fixed.

The main idea of the proposed method is that the largest Lyapunov exponent is computed based on one time series and used when the equations describing the system evolution are unknown and when it is impossible to measure all remaining phase coordinates.

Consider a time series x(t), t=1,…,N of one coordinate of a chaotic process measured in equal time intervals. The method of mutual information allows one to define the time delay τ, whereas the method of false neighbors yields the dimension of the embedded space *m*. As a result of the reconstruction, one gets a set of points of the space $R^{m}$:

(14) $$x_{i}=(x(i),x(i-\tau ),\ldots ,x(i-(m-1)\tau ))=(x_{1}(i),x_{2}(i),\ldots ,x_{m}(i)),$$

where i=((m−1)τ+1),…,N.

We take a point from the series (3) and denote it by $x_{0}.$ In the series (3), one can find a point ${\tilde{x}}_{0},$ where the relation $||{\tilde{x}}_{0}-x_{0}||=\varepsilon_{0}<\varepsilon$ holds, and where ε is a fixed quantity, essentially less than the dimension of the reconstructed attractor. It is required that the points $x_{0}$ and ${\tilde{x}}_{0}$ are separated in time. Then, time evolution of these points is observed on the reconstructed attractor until the distance between points achieves $\varepsilon_{\max}.$ The new points are denoted by $x_{1}$ and ${\tilde{x}}_{1},$ the distance is $\varepsilon_{0}^{\prime },$ and the associate interval of time evolution is denoted by $T_{1}$.

After that, we again consider the Sequence (14) the find the point ${\tilde{x}}_{1}^{\prime },$ located close to $x_{1},$ where $||{\tilde{x}}_{1}^{\prime }-x_{1}||=\varepsilon_{1}<\varepsilon$ holds. Vectors ${\tilde{x}}_{1}-x_{1}$ and ${\tilde{x}}_{1}^{\prime }-x_{1}$ should possibly have the same direction. Then, the procedure is repeated for points $x_{1}$ and ${\tilde{x}}_{1}^{\prime }$.

By repeating the above procedure *M* times, the largest Lyapunov exponent is estimated:

(15) $$\lambda ≅\sum_{k=0}^{M-1}\ln(\varepsilon_{k}^{\prime }/\varepsilon_{k})/\sum_{k=1}^{M}T_{k}.$$

This method has been employed in the present research to test the accuracy of results by using the classical and known spectra of the Lyapunov exponents of the Hénon map, Rössler equations, chaos and hyperchaos exhibited by the Lorenz system, and McKay-Glass equation [28]. In addition, it has been also employed to study the Belousov–Zhabotinsky reaction [29] and the Couette-Taylor flow [30].

Wolf et al. [1] have pointed out certain restrictions on the choice of the embedding dimension and the time required for the attractor reconstruction to achieve the most accurate estimates of the Lyapunov exponents. Using the Rössler attractor [16] and the Belousov–Zhabotynskiy reaction [29], the authors have demonstrated the effects of the time change during the attractor reconstruction, the time of evolution of the system between steps of the time change, the maximum length of the replacement vector and the minimum length of the exchange vector on the values of the estimated largest Lyapunov exponent. Furthermore, it has been shown that variation (between 0.5 and 1.5) of the time of the system evolution leads to reliable estimates of the studied three chaotic attractors. Also, some data requirements that make it possible to obtain the most accurate estimate of the Lyapunov exponent, such as the use of small length scale data as well as some restrictions on the presence of noisy perturbations in the data (static and dynamic), have been discussed.

The proposed algorithms can be used to detect chaos as well as to compute its parameters also for the experimental data with a few positive exponents. Furthermore, numerical studies have presented the topological complexity of chaos (the Lorenz attractor) and have shown that the deterministic chaos can be distinguished from white noise (the Belousov–Zhabotinsky reaction).

### 3.3. Rosenstein Method

Despite this method is simple in realization in comparison to the previous ones and it is characterized by high computational speed, it does not directly yield $\lambda_{1},$ but rather the function:

(16) $$y(i,\Delta t)=\frac{1}{\Delta t}\langle \ln d_{j}(i)\rangle ,d_{j}(i)=\min_{x_{j}}||x_{j}-x_{j}^{\prime }||,$$

where $x_{j}$ is a given point, and $x_{j}^{\prime }$ denotes its neighbor.

The algorithm is based on the relationship between $d_{j}$ and the Lyapunov exponents: $d_{j}(i)\approx e^{\lambda_{1}(i\Delta t)}.$ The largest Lyapunov exponent is computed by estimating the inclination of the most linear part of the function. It should be mentioned, however, that finding this linear part does not belong to easy tasks.

### 3.4. Kantz Method

The algorithm proposed by Kantz [5] computes the LLE by searching all neighbors in vicinity of the reference trajectory and estimates the average distance between neighbors and the reference trajectory as a function of time (or a relative time multiplied by the data sampling frequency). The algorithm is based on the following formula:

(17) $$S\left(\tau \right)=\frac{1}{T}\overset{T}{\underset{t=1}{\sum }}\ln\left(\frac{1}{\left|U_{t}\right|}\underset{i\in U_{t}}{\sum }\left|x_{t+\tau }-x_{i+\tau }\right|\right)$$

where $x_{t}$ stands for an arbitrary signal point; $U_{t}$ is a neighborhood of $x_{t}$; $x_{i}$ is a neighbor of $x_{t}$; τ—relative time multiplied by the sampling frequency; *T*—sample size; S(τ)—stretching factor in the region of a linear growth indicating a curve whose slope is equal to LE, i.e., $e^{\lambda \tau }\propto e^{S(\tau )}.$ However, the assumption of a linear growth introduces new errors. Despite the fact that the method is useful and accurate for systems with known LEs, the choice of parameters and the region where the mentioned linear growth occurs is, in practice, arbitrary.

The method yields correct results if the value of the Lyapunov exponent is known a priori, and hence the space with the tangent equal to that value can be chosen.

### 3.5. Computation of LLE Based on Synchronization of Nonnegative Feedback

In reference [7], the method of LLE computation based on synchronization of coupled identical systems has been proposed. The following *k*-dimensional discrete system:

(18) $$y_{i}^{\prime }=f\left(y_{i}\right)$$

has been considered, where $y\in {\mathbb{R}}^{k}$, i∈(1, 2, …, k). The supplemental system has been proposed in the following way:

(19) $$x_{i}^{\prime }=f\left(y_{i}+\Delta y_{i}\right)\;y_{i}^{\prime }=f\left(y_{i}\right)\;\Delta y_{i}^{\prime }=\left[f\left(y_{i}+\Delta y_{i}\right)-\;f\left(y_{i}\right)\right]\exp\left(-p\right)$$

where $x,\;y,\;\Delta y\in {\mathbb{R}}^{k}$. Evolution of *k*-dimensional system is governed by *k* of LLEs. Consequently, synchronization of the perturbed and nonperturbed systems (19) is guaranteed by the following inequality:

(20) $$p>\lambda_{\max}$$

where $\lambda_{\max}$ stands for LLEs of the studied systems (18).

**Figure 1:**
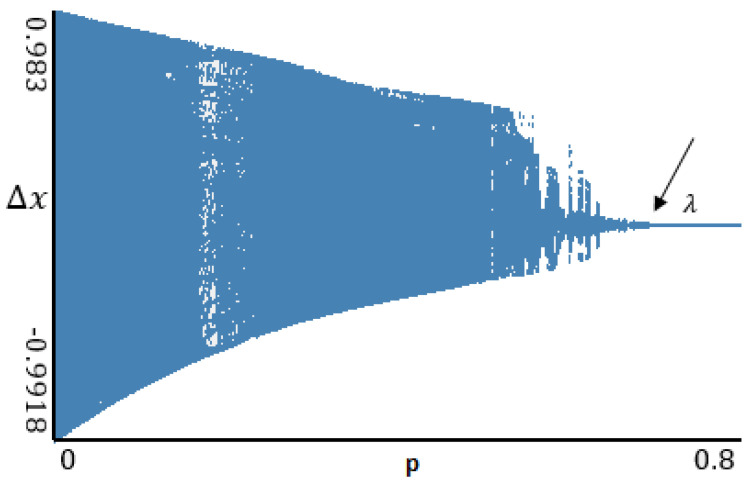
Synchronization of perturbed and nonperturbed systems in the case of a logistic map (λ points to the largest Lyapunov exponent value).

Figure 1 shows synchronization between perturbed (first equation of (19)) and nonperturbed (second equation of (19)) systems for alogistic map. The synchronization starts at *p* equal to λ, and this value represents the largest Lyapunov exponent of the system.

In reference [8], systems with excitations have been studied. The authors have proposed the following way of coupling of identical systems:

(21) $$\dot{x}=f\left(x\right)\;\dot{y}=f\left(y\right)+d\left(x-y\right)$$

The application of this approach is limited to the systems with known equations of evolutions, and the way of introducing the coupling of two identical systems depends on the type of the considered system.

### 3.6. Jacobi Method

**Figure 2:**
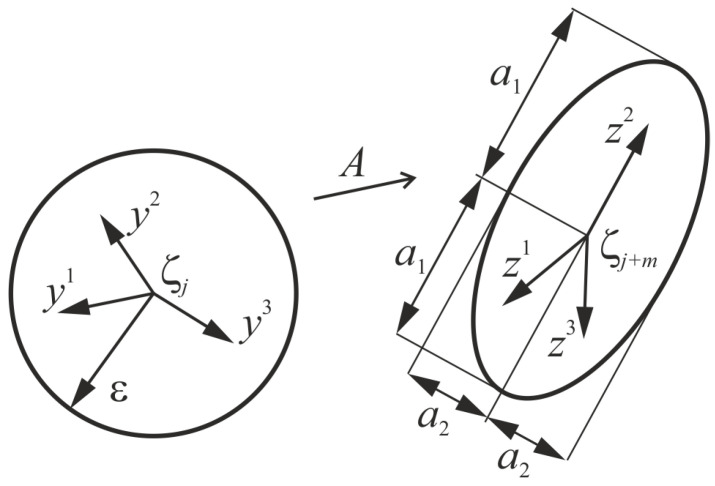
Transformation of a sphere of initial states into a counterpart ellipsoid during the system evolution.

This method has been proposed in references [31,32]. The main idea is to use an algorithm, the scheme of which is illustrated in Figure 2. A sphere of small radius ε is taken. After a few iterations *m*, a certain operator $T^{m}$ transforms this sphere into an ellipsoid having $a_{1},\ldots ,\;a_{p}$ half-axes. The sphere is stretched along the axes $a_{1},\ldots ,\;a_{s}>\varepsilon$, where *s* is the number of positive LEs. For sufficiently small ε, the operator $T^{m}$ is close to the sum of the shear operator and the linear operator *A*. The LLEs are computed as averaged eigenvalues of the operator *A* on the whole attractor.

A vector ${Ϛ}_{j}$ is chosen, and a set $\left\{{Ϛ}_{k_{i}}\right\}\left(i=1,\;\ldots ,\;N\right)$ of *i*-th neighborhood vectors is found. The following set of vectors $y_{i}\equiv {Ϛ}_{k_{i}}-{Ϛ}_{j}$, where $\|y_{i}\|\leq \varepsilon$, is taken. After *m* successive iterations, the operator $T^{m}$ transforms the vector ${Ϛ}_{j}$ into ${Ϛ}_{j+m}$, and the vector ${Ϛ}_{k_{i}}$ into ${Ϛ}_{k_{i+m}}$. Eventually, the vectors $y_{i}$ are transformed into

$$y_{i+m}={Ϛ}_{k_{i+m}}-{Ϛ}_{j+m}$$

Assuming that the radius ε is sufficiently small, one can introduce the operator $A_{j}$ as follows

$$y_{i+m}=A_{j}y_{i}$$

The operator $A_{j}$ describes the system in variations. To estimate the operator *A*, the least-square method can be employed:

$$\underset{A_{j}}{\min}S=\underset{A_{j}}{\min}\frac{1}{N}\overset{N}{\underset{i=0}{\sum }}{\left(y_{i+m}-A_{j}y_{i}\right)}^{2}$$

This yields the following system of equations of the dimension n×n:

$$A_{j}V=C,\;{\left(V\right)}_{kl}=\frac{1}{N}\overset{N}{\underset{i=1}{\sum }}y_{i}^{k}y_{i}^{l}$$

$${\left(C\right)}_{kl}=\frac{1}{N}\overset{N}{\underset{i=1}{\sum }}y_{i\;+\;m}^{k}y_{i}^{l}$$

where *V*, *C* are the matrices of the dimension n×n, $y_{i}^{k}$ stands for the *k*-th component of vector $y_{i}$, and $y_{i\;+\;m}^{k}$ is the *k*-th component of the vector $y_{i\;+\;m}$. If *А* is a solution of the equations, then the LEs can be found in the following way

$$\lambda_{i}=\underset{n\to \infty }{\lim}\frac{1}{n\tau }\overset{n}{\underset{j=1}{\sum }}\ln A_{j}e_{i}^{j}$$

where $\left\{e_{j}\right\}$ is a set of basic vectors in a tangent space ${Ϛ}_{j}$.

The algorithm can be realized in a way similar to the computation of LEs of the ODEs given analytically.

Let us choose an arbitrary basis $\left\{e^{s}\right\}$ and then follow the changes in the length of the vector $A_{j}e^{s}$. As the vectors $A_{j}e^{s}$ grow and their orientations change, it is necessary to perform their orthogonalization and normalization by using, for example, the Gramm–Schmidt procedure. The procedure is then repeated for the new basis.

The mentioned method allows one to estimate a spectrum of nonnegative LEs. However, it has a serious disadvantage—it is highly sensitive to noise and errors.

### 3.7. Modification of the Neural Network Method

**Figure 3:**
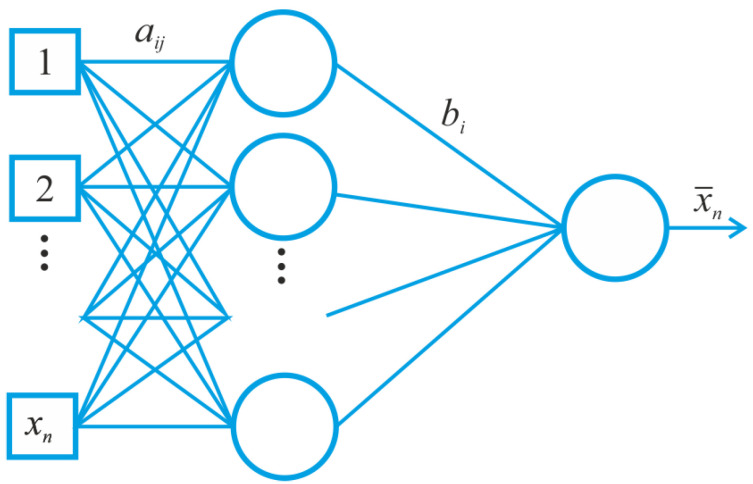
Single-layer feed forward neural network, which consists of input neurons, a layer of hidden neurons and one output neuron.

We have proposed a novel counterpart method to compute LEs based on a modification of the neural network method (see Figure 3).

A single-layer feed forward neural network presented in Figure 3 has multiple input neurons, a layer of hidden neurons and one output neuron. The following notation is employed: $a_{ij}$—weight of the connection between the *i*-th input neuron and the *j*-th hidden neuron; $b_{i}$—weight of connection between the *i*-th hidden neuron and the output neuron. To realize the neural network algorithm, the following criteria were taken into account:

(i) the network is sensitive to the input information (information is given in the form of real numbers);
(ii) the network is self-organizing, i.e., it yields the output space of solutions only based on the inputs;
(iii) the neural network is a network of straight distribution (all connections are directed from input neurons to output neurons);
(iv) owing to the synapses tuning, the network exhibits dynamic couplings (in the learning process, the tuning of the synaptic coupling takes place (dW/dt≠0), where *W* stands for the weighted coefficients of the network).

**Figure 4:**
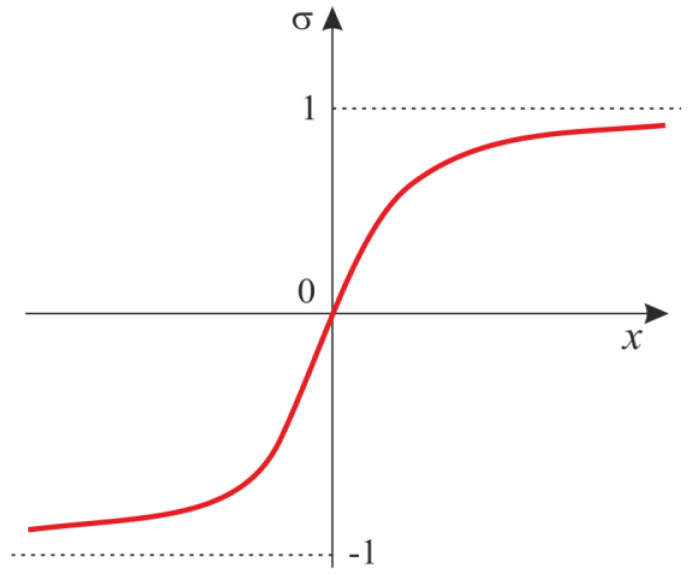
Transition function.

The hidden layer of neurons contains the hyperbolic tangent, which plays a role of an activation function (Figure 4). A derivative of the hyperbolic tangent is described by a quadratic function, as it is in the case of a logistic function. However, contrarily to the logistic function, the space of the values of the hyperbolic tangent falls within the interval (−1; 1). This results in higher convergence in comparison to the standard logistic function.

Prognosis of ${\hat{x}}_{k}$ of a scalar time series $x_{k}$ is made by employing the following formula

(22) $${\hat{x}}_{k}=\overset{n}{\underset{i=1}{\sum }}b_{i}\tanh\left(a_{i0}+\overset{d}{\underset{j=1}{\sum }}a_{ij}x_{k-j}\right)$$

where n stands for the number of neurons, d is the number of the searched LE, $a_{ij}$ stands for the n×(d+1) matrix of coefficients, and $b_{i}$ is the vector of the length n. The matrix $a_{ij}$ contains the coupling forces with respect to the network input, the vector $b_{i}$ is used to control the input of each neuron to the network output, whereas the vector $a_{i0}$ is used for relatively simple learning based on data with nonzero averaged value.

Weights a and b are chosen in a probabilistic way, and the dimension of the searched solution is decreased in the process of learning. The associated Gaussian is chosen in a way to have initial standard distribution $2^{-j}$, centered with respect to zero in order to promote the most recent time delays (small values of j) in the phase space. The coupling forces are chosen in a way to minimize the averaged one step mean square error of a forecast:

(23) $$e=\frac{\sum_{k=d+1}^{c}{\left({\hat{x}}_{k}-x_{k}\right)}^{2}}{c-d}$$

During the training of the network, sensitivity of the output is defined by computing partial derivatives of all averaged points of the time series in each time step $x_{k-j}$:

(24) $$\hat{S}\left(j\right)=\frac{1}{c-j}\overset{c}{\underset{k=j+1}{\sum }}\left|\frac{\partial {\hat{x}}_{k}}{\partial x_{k-j}}\right|$$

In the case of the network given by (22), the partial derivatives have the following form:

(25) $$\frac{\partial {\hat{x}}_{k}}{\partial x_{k-j}}=\overset{n}{\underset{i=1}{\sum }}a_{ij}b_{i}{\sec h}^{2}\left(a_{i0}+\overset{d}{\underset{m=1}{\sum }}a_{im}x_{k-m}\right)$$

The largest value j is the optimal embedding dimension, and the key role is played by $\hat{S}\left(j\right)$ as in the false nearest neighbors method. The individual values of $\hat{S}\left(j\right)$ yield a quantitative estimate of the importance of each time step using the associated terms of the autocorrelation function or coefficients of the associated linear model. 

The weights of the trained neural network are substituted to the matrix of solutions, and the input data are used to define the initial state. The computation of the spectrum is realized by employment of the generalized Benettin’s algorithm based on the obtained system of equations.

## 4. Wavelet Methods 

### Gauss Wavelets

In the majority of engineering problems, the Fourier analysis is insufficient, since it deals with the averaged spectrum of the whole studied vibration signal and presents only a general picture of the signal. On the contrary, wavelets play a role of a “microscope” which allows one to observe the spectrum at each time instant, and detect births/deaths of the frequencies in time.

A wavelet transform of a 1D signal is realized with respect to a basis being usually a soliton-like function with given properties. The basis is obtained by displacement and tension/compression of a function called a wavelet.

In the present work, the Gauss wavelets, defined as derivatives of the Gauss function, were used. Higher-order derivatives have many zero moments, and hence they allow one to obtain information about higher-order features hidden in the investigated signal.

The 8th order Gauss wavelets of the of the following form were employed:

(26) $$g_{8}\left(x\right)=-\left(105-\;420x^{2}+210x^{4}-28x^{6}+x^{8}\right)\exp^{\frac{-x^{2}}{2}}$$

## 5. Analysis of Classical Dynamical Systems by LEs and Gauss Wavelets

**Figure 5:**
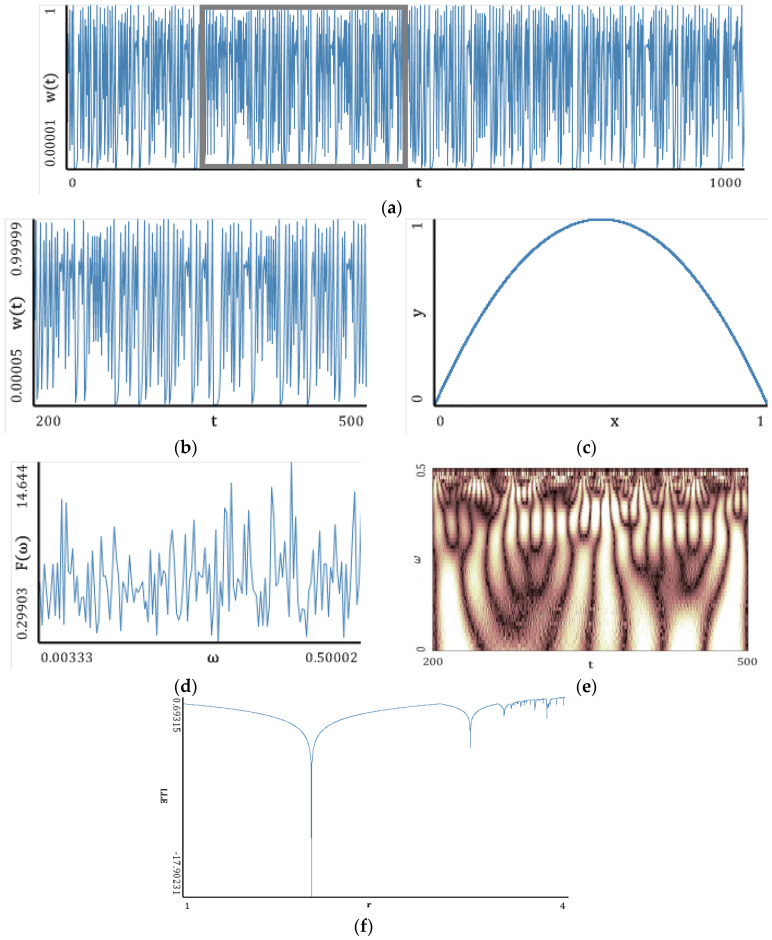
Nonlinear **c**haracteristics of the oscillation signal: (**a**) Time histories; (**b**) Time window; (**c**) Chaotic attractor; (**d**) Fourier frequency spectrum; (**e**) Wavelet spectrum; (**f**) Dependence of LLE on the control parameter.

**Figure 6:**
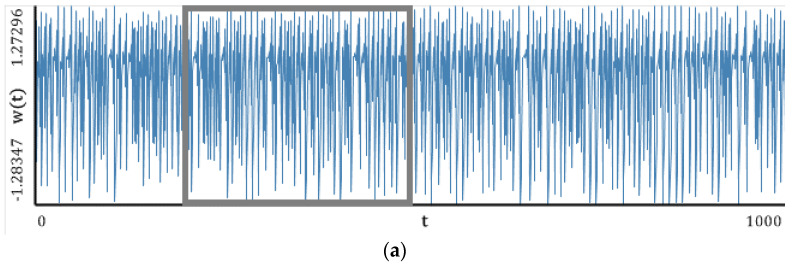

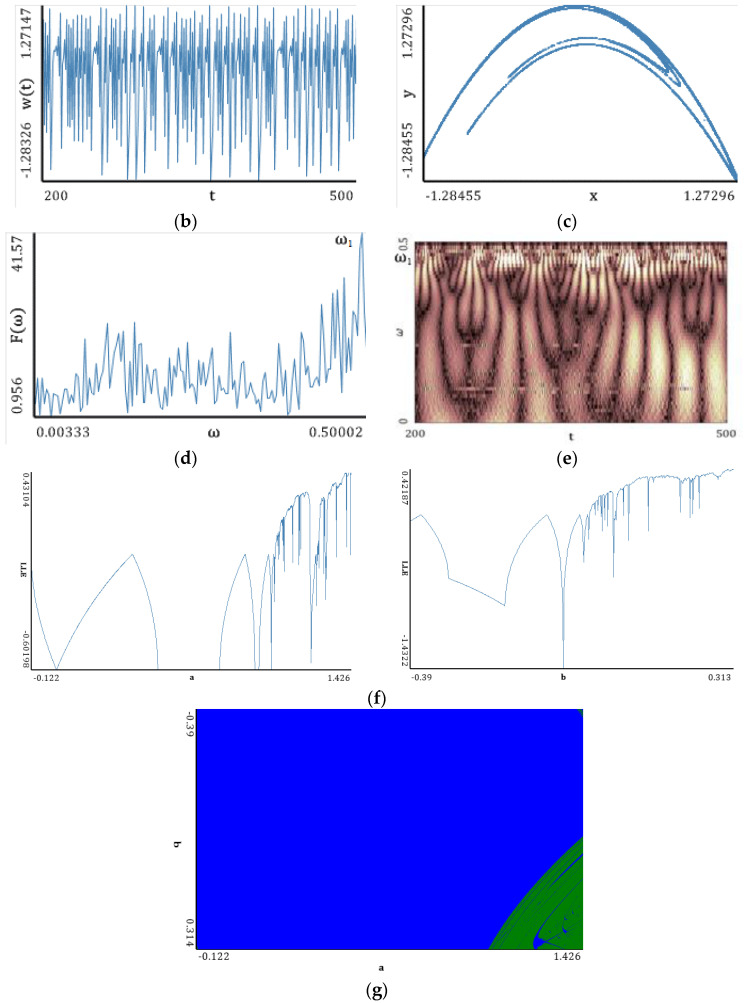
Characteristics of the Hénon map: (**a**) Time history; (**b**) Time window; (**c**) Chaotic attractor; (**d**) Fourier frequency spectrum; (**e**) Wavelet spectrum; (**f**) Dependence of LLE on the control parameter; (**g**) Lyapunov exponents plane (Hénon map).

**Figure 7:**
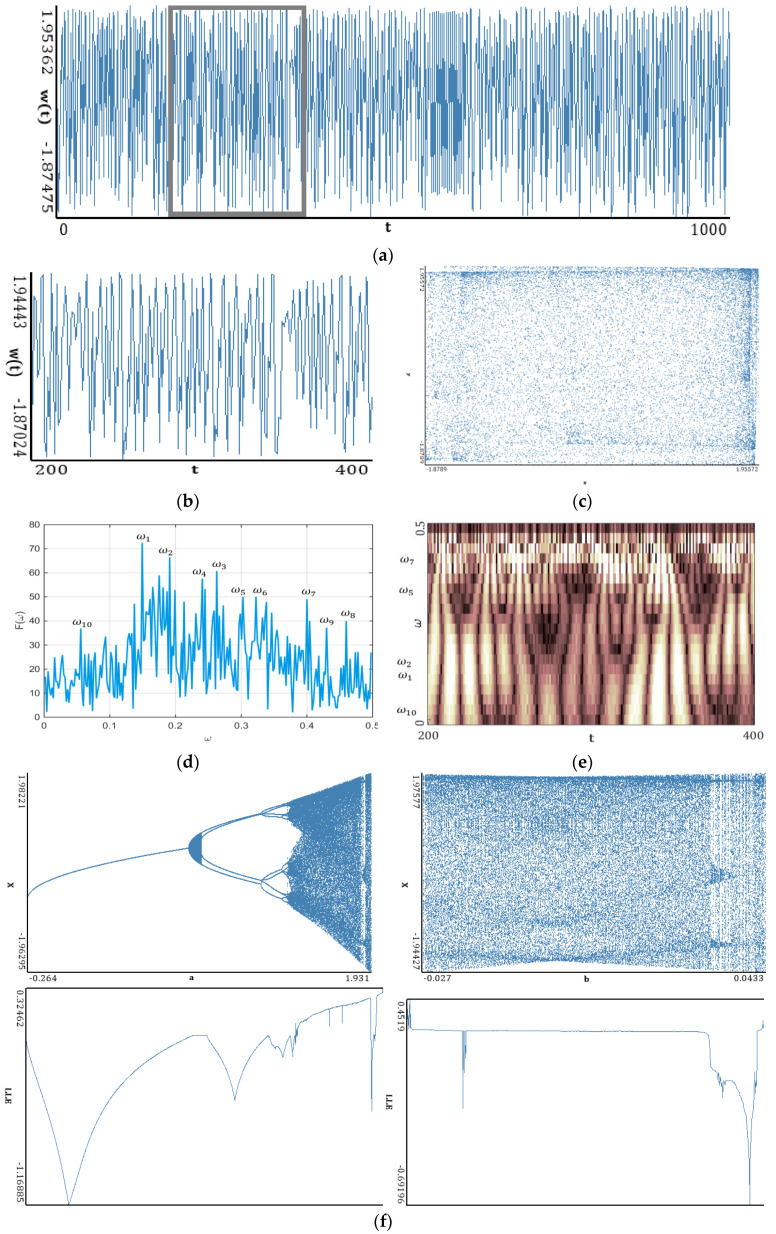

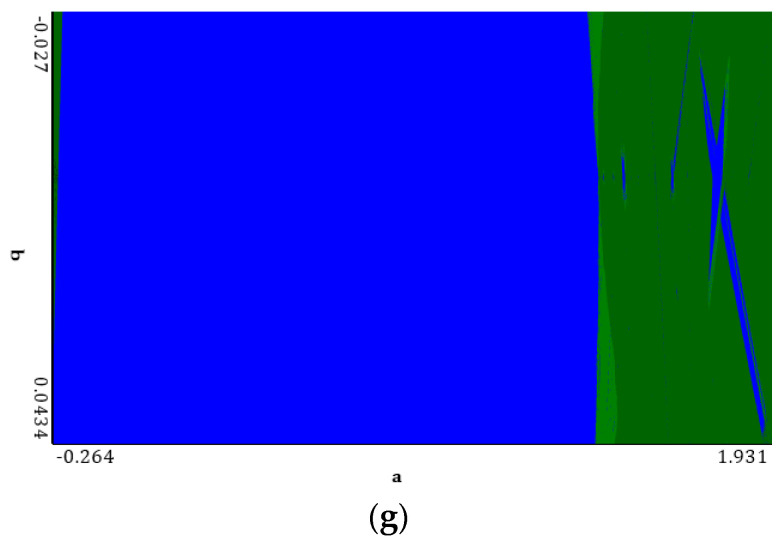
Signal characteristics: (**a**) Time history; (**b**) Time window; (**c**) Chaotic attractor; (**d**) Fourier frequency spectrum; (**e**) Wavelet spectrum; (**f**) Dependence of LLE on the control parameter; (**g**) Lyapunov exponents plane (generalized Hénon map).

**Figure 8:**
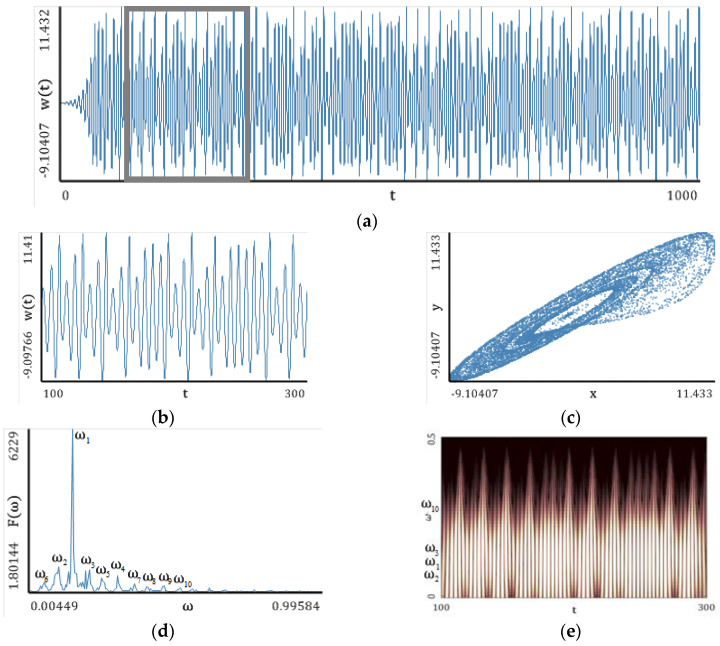

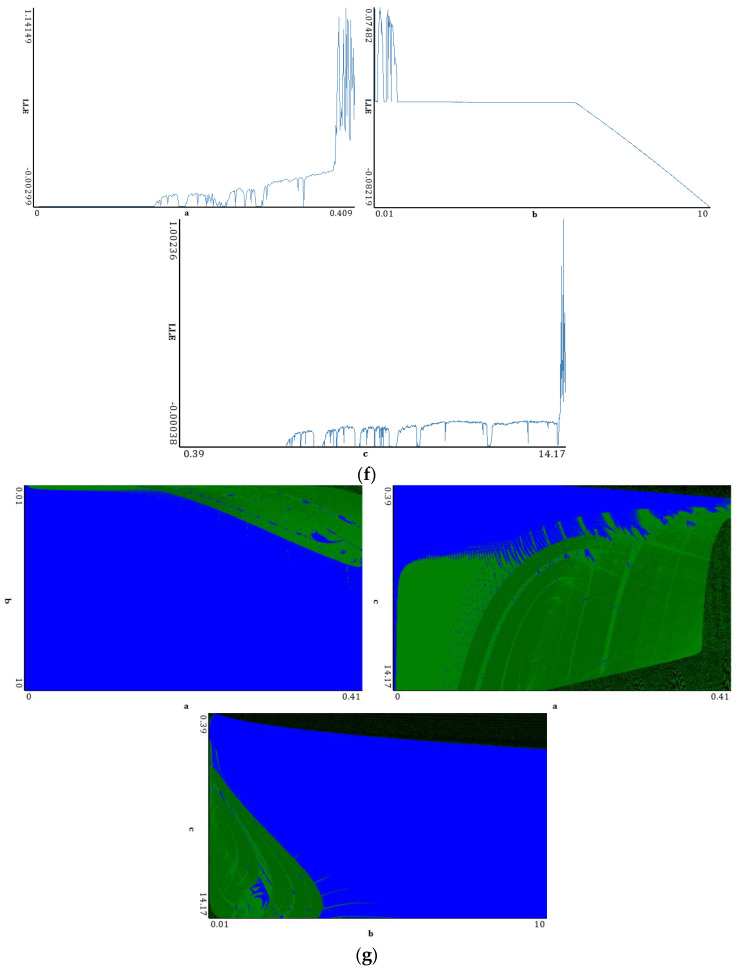
Signal characteristics: (**a**) Time history; (**b**) Time window; (**c**) Chaotic attractor; (**d**) Fourier frequency spectrum; (**e**) Wavelet spectrum; (**f**) Dependence of LLE on the control parameter; (**g**) Lyapunov exponents plane (Rössler attractor).

**Figure 9:**
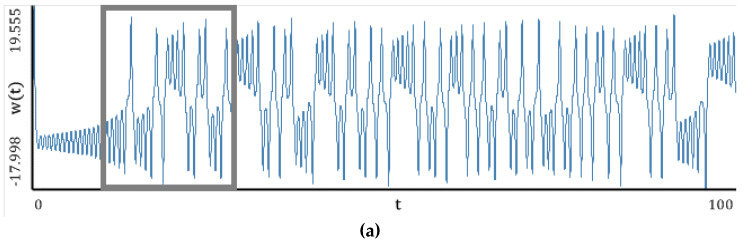

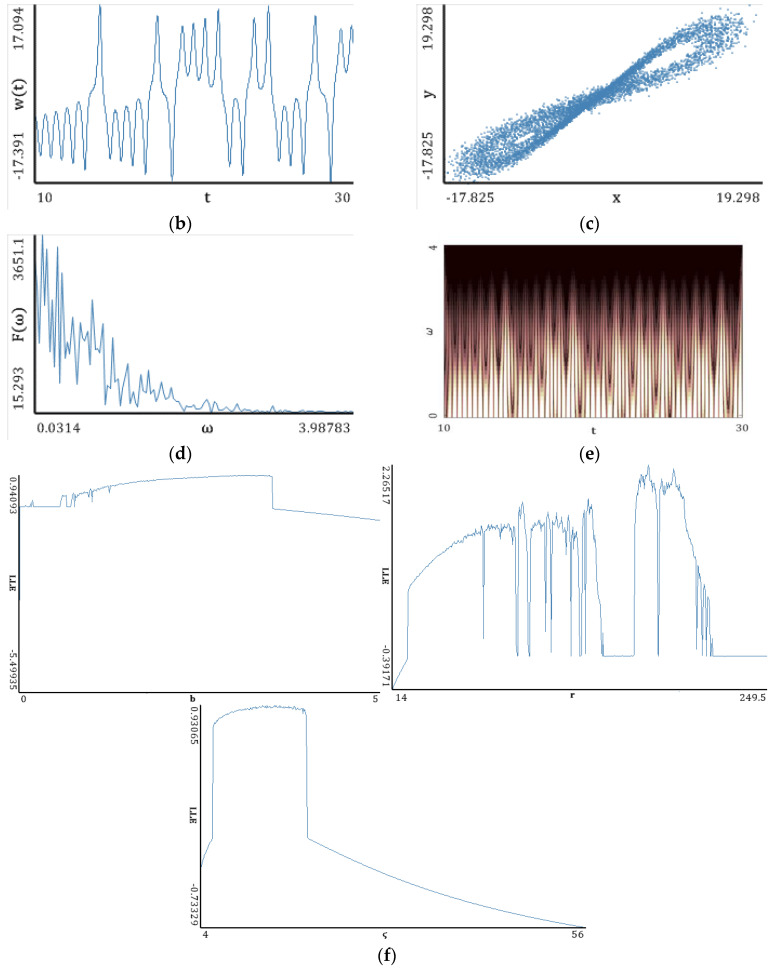

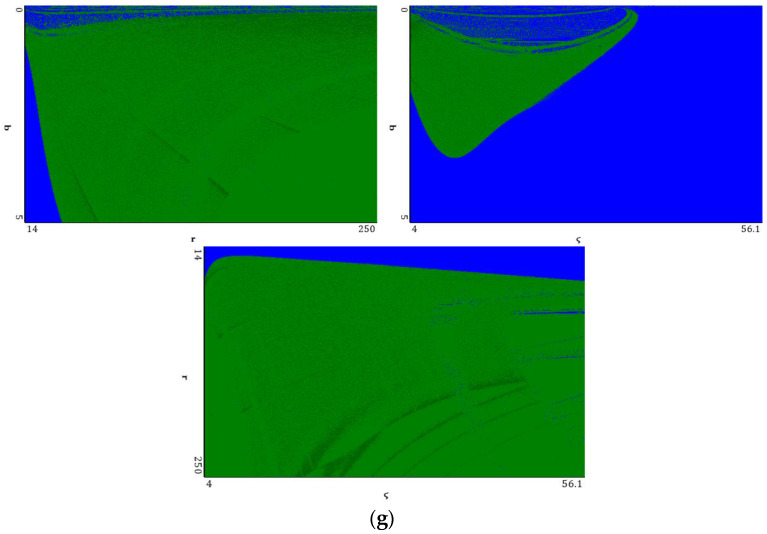
Signal characteristics: (**a**) Time history; (**b**) Time window; (**c**) Chaotic attractor; (**d**) Fourier frequency spectrum; (**e**) Wavelet spectrum; (**f**) Dependence of LLE on the control parameter; (**g**) Lyapunov exponents plane (Lorenz attractor).

In this section, simple classical systems (Figure 5, Figure 6, Figure 7, Figure 8 and Figure 9) have been studied with emphasis put on a comparison of the LEs (Table 1, Table 2, Table 3, Table 4 and Table 5) obtained using the Wolf, Rosenstein, Kantz and neural network methods. The convergence of the mentioned methods, depending on the number of iteration steps, has been illustrated and discussed (Table 6, Table 7, Table 8, Table 9 and Table 10). The Benettin method has been used as a reference because for most systems, there are no analytically calculated spectra of Lyapunov exponents. Moreover, the Benettin method calculates Lyapunov exponents based on the system equations.

### 5.1. Logistic Map

A logistic map describes how the population changes with respect to time:

(27) $$X_{n+1}=RX_{n}\left(1-X_{n}\right)$$

Here, $X_{n}$ takes the values from 0 to 1 and presents the population in the *n*-th year, whereas $X_{0}$ denotes the initial population (in the year 0); *R* is a positive parameter characterizing an increase in the population (computations were carried out for *R* = 4). The first Lyapunov exponent and the Kaplan–Yorke dimension have been estimated by Sprott [33,34]. He has obtained: *λ*_1_ = 0.693147181, and the Kaplan–Yorke dimension: 1.0. 

Figure 5, Figure 6, Figure 7, Figure 8 and Figure 9 report the following results: (а) signal; (b) signal window; (c) chaotic attractor; (d) Fourier power spectrum; (e) Gauss wavelet of the 8th order, described in Section 4; (f) LLE change depending on the system control parameter; (g) LEs on the control parameters plane (where: 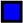
—only negative Lyapunov exponents, 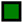
—one positive exponent, 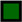
—two positive exponents, 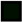
—three positive exponents).

The power spectrum is noisy and it is not possible to distinguish the dominating frequency. A similar situation is exhibited by the Gauss wavelet, where a large set of frequencies is visible. Dynamics of LLE changing increases for *r* > 3. 

As can be seen in Table 1, all computational methods were compared with Benettin’s original results. Good coincidence was exhibited by the neural network method, the Rosenstein method, the Kantz method, and the method of synchronization. The Wolf method gave decreased/increased value of LLE in comparison to the original value.

### 5.2. Hénon Map

The Hénon map takes a point $\left(X_{n},Y_{n}\right)$ and maps it into another point by the following formulas:

(28) $$\begin{array}{l} X_{n+1}=1-aX_{n}^{2}+Y_{n},\\ Y_{n+1}=bX_{n}. \end{array}$$

The following parameters are fixed for numerical experiments: a=1.4, b=0.3. Since the Equations (28) do not correspond to a real object, the parameters are replaced with fixed values. Sprott [34] has computed the Lyapunov spectrum and the Kaplan–Yorke dimension of the map using the Benettin method [27] by solving (28). He has obtained the following LEs: $\lambda_{1}=0.419217,$ $\lambda_{2}=-1.623190$, and the Kaplan–Yorke dimension: 1.258267.

Similarly to the logistic map, the power spectrum exhibits a uniform noisy shape. However, one can distinguish a dominating frequency ($\omega_{1}\approx 0,45$). This frequency is also visible on the wavelet spectrum as a region of the largest amplitudes along the whole signal (brighter regions in the graph). Plots of the change in the LLE correlate with bifurcation diagrams for the same interval of changes in the parameters *a* and *b*. Dynamics of the LLE changes increases with the increase in both control parameters. Starting with the graphs of LEs for a given set of control parameters, the system mainly remains in a periodic regime, but it exhibits chaotic dynamics for large values of the control parameters.

Beginning from the results shown in Table 2, the majority of the employed computational methods yielded good results. However, the most accurate results were obtained by the neural network method (for whole spectrum of LEs), the Rosenstein method, the Kantz method, and the method of synchronization (in the case of LLEs). The Wolf method gave decreased estimated values of the LLEs.

### 5.3. Hyperchaotic Generalised Hénon Map

To obtain the hyperchaotic Hénon map, one needs to take a point $\left(X_{n},Y_{n},Z_{n}\right)$ and map it into the following one:

(29) $$\begin{array}{l} X_{n+1}=a-aY_{n}^{2}-bZ_{n},\\ Y_{n+1}=X_{n},\\ Z_{n+1}=Y_{n}. \end{array}$$

The computations were carried out for the following fixed parameters: a=3.4, b=0.1. The Lyapunov spectrum reported in reference [14] is: 0.276; 0.257; 4.040.

One can distinguish a large number of frequencies in the power spectrum. Frequencies with the largest amplitude are located in the interval [0.15; 0.3] (frequencies $\omega_{1}-\omega_{4}$), but the remaining part of the spectrum is noisy. This interval corresponds to the brightest region on the Gauss wavelet, which is correlated with the values of the power spectrum. Changes in LLEs coincide with the bifurcation diagrams constructed for the same intervals of changes in the control parameters *a* and *b*. Dynamics of LLEs increases with the increase in the control parameters. As in the case of the Hénon map, the chart of LEs for the selected control parameters exhibits, for a majority of studied parameters, periodic dynamics. It transits into chaos for a≈1.4, and is almost suddenly shifted into hyperchaos (2 positive LEs).

Good results were obtained by the Benettin, Rosenstein and synchronization methods (divergence from the third decimal place). The neural network yielded slightly increased estimates of two first LEs, whereas the third LE was estimated almost exactly. The Kantz method gave a decreased result in comparison to reference data. The Wolf method resulted in the largest error.

### 5.4. Rössler Attractor

The following Rössler system of ODEs was investigated:

(30) $$\{\begin{array}{c} \dot{x}=-y-z,\\ \dot{y}=x+ay,\\ \dot{z}=b+z(x-c), \end{array}$$

and the computations were carried out for the following fixed parameters a=b=0.2 and c=5.7. The original study yielded the Lyapunov spectrum: 0.0714, 0, −5:3943, and the Kaplan–Yorke dimension equal to 2.0132.

The power spectrum contains the fundamental frequency $\omega_{1}$, which is accompanied by damped bursts (frequencies $\omega_{2}-\omega_{10}$). In the whole time interval, the Gauss wavelet exhibits the brightest region of the fundamental frequency with darker peaks going to zero. Thus, the picture is analogous to the power spectrum. Contrarily to the studied maps, the bifurcation diagrams have a more complex structure. However, there is still correlation with the changes in LLEs for the corresponding control parameters. The parameter *b* has the smallest influence on the change in LLE. Graphs of LLEs also exhibit a more complex structure. Borders of different vibration kinds have complex forms, which illustrates the increase in the system complexity. Aside from the chaos and hyperchaos zones, there are drops indicating 3 positive LEs. Amabili et al. [35] have suggested to call all chaotic oscillations, for which at least two positive Lyapunov exponents exist, by hyperchaotic.

As far as Table 4 is considered, the best results were yielded by the Benettin and Rosenstein methods. The method of neural networks gave very good results in the case of estimates of two first LEs, but underestimated the third exponent. The Wolf method yielded smaller value of the first exponent compared to the reference data. The most underestimated results were given by the Kantz method.

The carried out numerical experiments showed that changing the sampling frequency did not affect the power spectrum and wavelet spectrum. This was also validated by results obtained by the Benettin, neural networks, and Rosenstein methods, which yielded the results very close to original ones. The Kantz method gave underestimated results for different sampling frequency, correlating with the results obtained for the standard sample size. 

### 5.5. Lorenz Attractor

The system is described by the following ODEs:

(31) $$\{\begin{array}{c} \dot{x}=\sigma (y-x),\\ \dot{y}=x(r-z)-y,\\ \dot{z}=xy-bz, \end{array}$$

where *r* stands for the normalized Rayleigh number (nondimensional number defining fluid behavior under gradient):

(32) $$r=\frac{g\beta \Delta TL^{3}}{\nu \chi }.$$

In the above equation, the following notation is used: *g*—gravity of Earth; *L*—characteristic dimension of the fluid space; ΔT—temperature difference between fluid walls; ν—kinematic fluid viscosity, χ—thermal conductivity of the fluid; β—coefficient of heat fluid extension; σ—Prandtl number (takes into account heat source property) governed by the following equation

(33) $$\sigma =\frac{\nu }{\alpha }=\frac{\eta C_{p}}{ℵ},$$

where: ν=η/ρ—kinematic viscosity, η—dynamic viscosity, ρ—density, $\alpha =\frac{ℵ}{\rho C_{p}}$—temperature transfer coefficient, ℵ—heat transfer coefficient, $C_{p}$—specific heat capacity under constant pressure; and ρ—information about the geometry of the convective cell. 

The following parameters were fixed: σ=10.0, r=28.0, b=8/3. The original results are: LEs: 0.9056, 0, −14.5723; the Kaplan–Yorke dimension: 2.06215.

The power spectrum of the attractor decreases uniformly when approaching a finite frequency, and there are no frequencies with a strongly dominating amplitude. The latter observation has been also verified by the Gauss wavelet spectrum. The bifurcation diagrams, similar to those for the Rössler system, exhibit a complex structure, but the correlation to the LLEs change is conserved. The richest/lowest dynamics of LLE is obtained for changing parameter r/σ. Based on the reported graphs of LEs, one can conclude that the system dynamics is fully chaotic. There are also narrow windows of hyperchaotic dynamics.

A comparison of the results reported in Table 5 with the original results exhibits an excellent coincidence of the Benettin method (original results) and the neural network method (+4.79%). The Wolf and Rosenstein methods yielded the underestimated results of the LLE value. The worst estimation has been obtained by Kantz method.

Changing the sampling frequency did not change Fourier and wavelet power spectra. This was also validated by the Benettin and Rosenstein methods, which yield the results very close to the original values in spite of the arbitrary choice of the sampling frequency.

## 6. Concluding Remarks

The analysis of the dynamics of the studied classical system by different methods leads to a conclusion that the most perspective and useful is the modified method of neural networks [9,10]. It gives excellent convergence to the original results and, as the only one (besides of the Benettin method), allows to compute the spectrum of all Lyapunov exponents. In addition, very good results were obtained by the Rosenstein and Kantz methods for all studied systems. However, this method can be used to estimate only the largest Lyapunov exponents. 

As far as the convergence is considered, the Wolf method yielded either over- or underestimated values of LEs. The method of synchronization worked reasonably well for the maps, but it was not useful in studying differential equations (the Rössler or Lorenz systems). The mentioned systems require the use of another type of coupling, which is a drawback of the method.

It should be emphasized that this part of the paper serves as a preliminary study of a more complicated nonlinear continuous structural system, which is studied in Part 2. The carried out analysis of the works devoted to feasible methods for computation of Lyapunov exponents shows that there is no universal, verified, and general method to compute the exact (in the sense of numerics) values of the Lyapunov exponents. This observation leads to the conclusion that there is a need to employ qualitatively different methods to check the reliability of “true chaotic results”. Furthermore, the carried out analysis is a helping tool to study systems of an infinite dimension. Such an analysis is the subject of the second part of the paper.

## Figures and Tables

**Table 1:** Spectrum of Lyapunov exponents and LLEs computed by different methods (logistic map).

**LE Spectrum**			
Benettin Method		Neural Network	
(LEs): 0.69315 Dimension Kaplan–Yorke (DKY): 1 Kolmogorov-Sinai entropy (KSE): 0.69315 Phase volume compression (PVC): 0.69315		LEs: 0.69290 DKY: 1 EKS: 0.69290 PVC: 0.69290	
**LLE**			
Wolf Method	Rosenstein Method	Kantz Method	Method of Synchronization
LLE: 0.99683	LLE: 0.690553	LLE: 0.69810	LLE: 0.696

**Table 2:** Lyapunov exponents spectrum and LLEs computed by different methods (Hénon map).

**Spectrum of LLEs**			
Benettin Method		Neural Network	
LEs: 0.41919; −1.62316 DKY: 1.25826 EKS: 0.41919 PVC: −1.20397		LEs: 0.41919; −1.62316 DKY: 1.25826 EKS: 0.41919 PVC: −1.20397	
**LLEs**			
Wolf Method	Rosenstein Method	Kantz Method	Synchronization Method
LLE: 0.38788	LLE: 0.414218	LLE: 0.41912	LLE: 0.40608

**Table 3:** Lyapunov exponents spectrum and LLEs computed by different methods (generalized Hénon map).

**Spectrum of LEs**			
Benettin Method		Neural Network	
LEs: 0.27628; 0.25770; −4.04053 DKY: 2.13215 EKS: 0.53397 PVC: −3.50656		LEs: 0.29251; 0.27104; −4.04583 DKY: 2.13929 EKS: 0.56355 PVC: −3.48227	
**LLEs**			
Wolf Method	Rosenstein Method	Kantz Method	Synchronization Method
LLE: 0.45214	LLE: 0.27930	LLE: 0.26601	0.27250

**Table 4:** Lyapunov exponents spectrum and LLEs computed by different methods (Rössler attractor).

**Spectrum of LEs**		
Benettin Method	Neural Network	
LE: 0.07135; 0.00000; −5.39420 DKY: 2.01323 KSE: 0.07135 PVC: −5.32285	LE: 0.07593; −0.00060; −0.78178 DKY: 2.09635 EKS: 0.07593 PVC: −0.70646	
**LLEs**		
Wolf Method	Rosenstein Method	Kantz Method
LLE: 0.05855	LLE: 0.0726	LLE: 0.0774

**Table 5:** Lyapunov exponents spectrum and LLEs computed by different methods (Lorenz attractor).

**Spectrum of LEs**		
Benettin Method	Neural Network Method	
LE: 0.90557; 0.00000; −14.57214 DKY: 2.06214 EKS: 0.90557 PVC: −13.66656	LE: 0.9490; 0.0610; −13.9101 DKY: 2.07261 EKS: 1.0101 PVC: −12.9000	
**LLEs**		
Wolf Method	Rosenstein Methhod	Kantz Method
LLE: 0.81704	LLE: 0.836	LLE: 0.807185

**Table 6:** Fourier power spectra (**a**) and Gauss wavelet spectra (**b**) obtained for Δt=1, 2 and the LLEs computed by different methods (logistic map).

Δt=1	Δt=2
Fourier Power Spectra (**a**)	
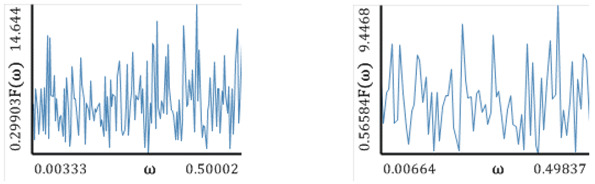	
Gauss Wavelet Spectra (**b**)	
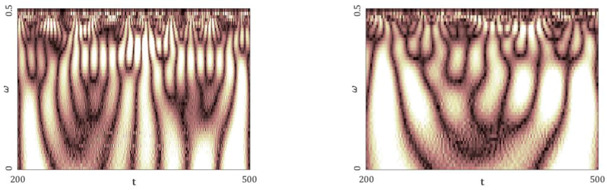	
LLE (Wolf)	
0.99961	1.00014
LLE (Rosenstein)	
0.69231	0.69065
LLE (Kantz)	
0.6981	0.69005
LLE (Synchronization)	
0.69400	0.69330
LEs (Benettin)	
LES: 0.69318 DKY: 1.00000 KSE: 0.69318 PVC: 0.69318	LES: 0.69400 DKY: 1.00000 KSE: 0.69400 PVC: 0.69400
LEs (Neural Network)	
LES: 0.69290 DKY: 1 SE: 0.69290 PVC: 0.69290	LES: 0.69107 DKY: 1.00000 KSE: 0.69107 PVC: 0.69107

**Table 7:** Fourier power spectra (**a**) and Gauss wavelet spectra (**b**) obtained for Δt=1, 2 and the computed LLEs by different methods (Hénon map).

Δt=1	Δt=2
Fourier Power Spectra (**a**)	
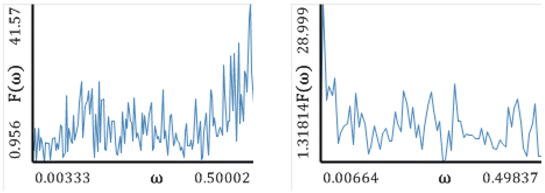	
Gauss Wavelet Spectra (**b**)	
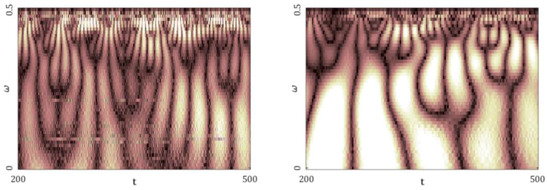	
LLE (Wolf)	
0.4158	0.39734
LLE (Rosenstein)	
0.41637	0.400635
LLE (Kantz)	
0.41912	0.41478
LLE (Synchronization)	
0.40608	0.40510
All LEs (Benettin)	
LEs: 0.41919; −1.62316 DKY: 1.25826 EKs: 0.41919 PVC: −1.20397	LEs: 0.41917; −1.62315 DKY: 1.25825 EKs: 0.41917 PVC: −1.20397
All LEs (Neural Network)	
LEs: 0.41919; −1.62316 DKY: 1.25826 KSE: 0.41919 PVC: −1.20397	LEs: 0.40924; −1.61321 DKY: 1.25368 KSE: 0.40924 PVC: −1.20397

**Table 8:** Fourier power spectra (**a**) and Gauss wavelet spectra (**b**) obtained for Δt=1, 2 and the computed LLEs by different methods (generalized Hénon map).

Δt=1	Δt=2
Fourier Power Spectra (**a**)	
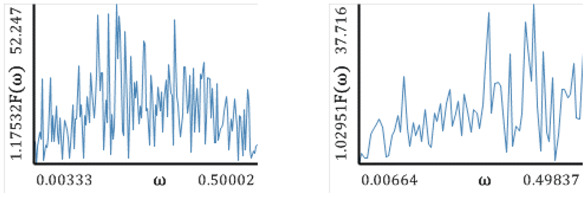	
Gauss Wavelet Spectra (**b**)	
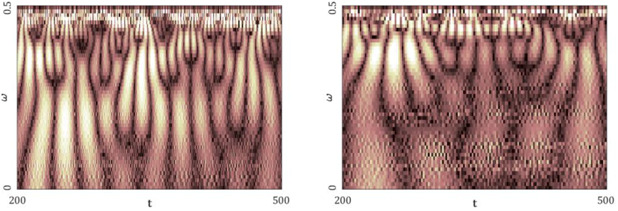	
LLE (Wolf)	
0.45214	0.46706
LLE (Rosenstein)	
0.27930	0.27459 (0.62515)
LLE (Kantz)	
0.26601	0.3359
LLE (Synchronization)	
0.27250	0.27200
All LEs (Benettin)	
LEs: 0.27628; 0.25770; −4.04053 DKY: 2.13215 KSE: 0.53397 PVC: −3.50656	LEs: 0.27487; 0.25631; −4.03774 DKY: 2.13155 EKS: 0.53118 PVC: −3.50656
All LEs (Neural Network)	
LEs: 0.29251; 0.27104; −4.04583 DKY: 2.13929 KSE: 0.56355 PVC: −3.48227	LEs: 0.26304; 0.24387; −4.14321 DKY: 2.12235 KSE: 0.50691 PVC: −3.63630

**Table 9:** Fourier power spectra and Gauss wavelet spectra obtained for Δt=0.05, 0.1, 0.15, 0.2 and the computed LLEs by different methods **(**Rössler attractor).

Δt=0.05	Δt=0.1	Δt=0.15	Δt=0.2
Fourier Power Spectrum			
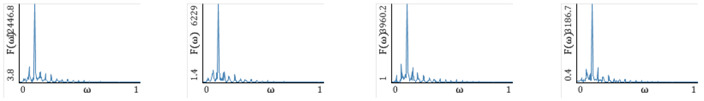			
Gauss Wavelets			
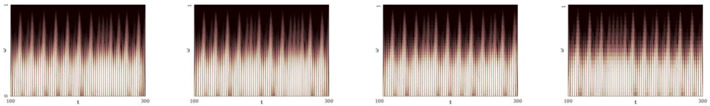			
LLE (Wolf)			
0.07283	0.05855	0.01731	0.02544
LLE (Rosenstein)			
0.083	0.0726	0.06553	0.606
LLE (Kantz)			
0.0234	0.0208	0.02133	0.0215
All LEs (Benettin)			
LES: 0.07156; 0.00000; −5.38768 DKY: 2.01328 KSE: 0.07156 PVC: −5.31612	LES: 0.06959; 0.00000; −5.21949 DKY: 2.01333 KSE: 0.06959 PVC: −5.14990	LES: 0.06789; 0.00000; −4.34385 DKY: 2.01563 KSE: 0.06789 PVC: −4.27596	LES: 0.06205; −0.00001; −2.84111 DKY: 2.02184 KSE: 0.06205 PVC: −2.77906
All LEs (neural network)			
LES: 0.06259; −0.07984; −0.32528 DKY: 1.78396 KSE: 0.06259 PVC: −0.34253	LES: 0.07340; −0.02681; −0.23525 DKY: 2.19807 KSE: 0.07340 PVC: −0.18865	LES: 0.07374; 0.00057; −0.36909 DKY: 2.20135 KSE: 0.07432 PVC: −0.29477	LES: 0.07983; −0.02816; −0.91182 DKY: 2.05667 KSE: 0.07983 PVC: −0.86015

**Table 10:** Fourier power spectra and Gauss wavelet spectra obtained for Δt=0.005, 0.01, 0.015, 0.02 and the computed LLEs by different methods (Lorenz attractor).

Δt=0.005	Δt=0.01	Δt=0.015	Δt=0.02
Fourier Power Spectrum			
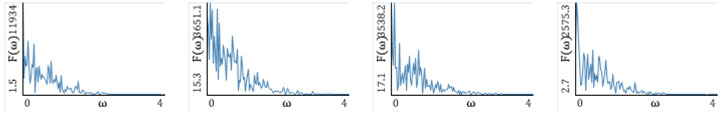			
Gauss Wavelet			
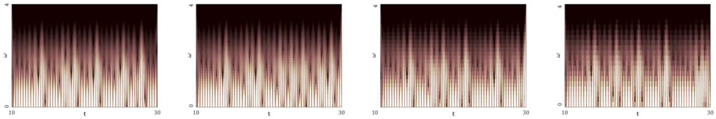			
LLE (Wolf)			
0.9721	0.81704	0.867	0.712
LLE (Rosenstein)			
0.876	0.836	0.858	0.859
LLE (Kantz)			
0.898	0.9	0.762667	0.84
LES (Benettin)			
LES: 0.90632; 0.00000; −14.57297 DKY: 2.06219 KSE: 0.90632 PVC: −13.66666	LES: 0.90523; 0.00000; −14.57179 DKY: 2.06212 KSE: 0.90523 PVC: −13.66656	LES: 0.90551; 0.00000; −14.57163 DKY: 2.06214 KSE: 0.90551 PVC: −13.66613	LES: 0.90596; 0.00000; −14.57086 DKY: 2.06218 KSE: 0.90596 PVC: −13.66490
LES (Neural Network)			
LES: 0.91677; 0.04404; −6.464 DKY: 2.14864 EKS entropy: 0.96081 PVC: 0.89617	LE: 0.9490; 0.0610; −13.9101 DKY: 2.07261 EKS: 1.0101 PVC: −12.9000	LES: 0.8913; −0.3508; −14.3577 DKY: 2.03765 EKS: 0.8913 PVC: −13.8172	LES: 0.7485; −0.05558; −23.3505 DKY: 2.0296 EKS: 0.7485 PVC: −22.65758
